# Preference-based measures to obtain health state utility values for use in economic evaluations with child-based populations: a review and UK-based focus group assessment of patient and parent choices

**DOI:** 10.1007/s11136-018-1831-6

**Published:** 2018-03-21

**Authors:** Jane L. Wolstenholme, Danielle Bargo, Kay Wang, Anthony Harnden, Ulla Räisänen, Lucy Abel

**Affiliations:** 1 Health Economics Research Centre, Nuffield Department of Population Health, University of Oxford, New Richards Building, Old Road Campus, Oxford, OX3 7LF UK; 2 Nuffield Department of Primary Care Health Sciences, University of Oxford, Oxford, UK

**Keywords:** Preference-based measures, Quality of life, Children, Utility, EQ-5D, CHU-9D

## Abstract

**Background:**

No current guidance is available in the UK on the choice of preference-based measure (PBM) that should be used in obtaining health-related quality of life from children. The aim of this study is to review the current usage of PBMs for obtaining health state utility values in child and adolescent populations, and to obtain information on patient and parent–proxy respondent preferences in completing PBMs in the UK.

**Methods:**

A literature review was conducted to determine which instrument is most frequently used for child-based economic evaluations and whether child or proxy responses are used. Instruments were compared on dimensions, severity levels, elicitation and valuation methods, availability of value sets and validation studies, and the range of utility values generated. Additionally, a series of focus groups of parents and young people (11–20 years) were convened to determine patient and proxy preferences.

**Results:**

Five PBMs suitable for child populations were identified, although only the Health Utilities Index 2 (HUI2) and Child Heath Utility 9D (CHU-9D) have UK value sets. 45 papers used PBMs in this population, but many used non-child-specific PBMs. Most respondents were parent proxies, even in adolescent populations. Reported missing data ranged from 0.5 to 49.3%. The focus groups reported their experiences with the EQ-5D-Y and CHU-9D. Both the young persons’ group and parent/proxy groups felt that the CHU-9D was more comprehensive but may be harder for a proxy to complete. Some younger children had difficulty understanding the CHU-9D questions, but the young persons’ group nonetheless preferred responding directly.

**Conclusion:**

The use of PBMs in child populations is increasing, but many studies use PBMs that do not have appropriate value sets. Parent proxies are the most common respondents, but the focus group responses suggest it would be preferred, and may be more informative, for older children to self-report or for child–parent dyads to respond.

**Electronic supplementary material:**

The online version of this article (10.1007/s11136-018-1831-6) contains supplementary material, which is available to authorized users.

## Background

In the 2013 NICE guide to Methods of Technology Appraisals, the EQ-5D was indicated to be the preferred preference-based measure (PBM) to obtain utility values in adults. However, in terms of children it was stated:

“Consideration should be given to alternative standardized and validated preference-based measures of health-related quality of life that have been designed specifically for use in children. The standard version of the EQ-5D has not been designed for use in children. An alternative version for children aged 7–12 years is available, but a validated UK valuation set is not yet available” [1].

The NICE guidelines do not specify the use of a PBM that had been designed for use in children, nor mention at what age it is appropriate or inappropriate for a child to respond to a PBM. As a result, there is currently a void in terms of guidance on which instruments are appropriate and when it is appropriate for a child to respond to a PBM.

Several studies have been conducted on the topic of utility values and children. An overview of issues to consider when undertaking pediatric economic evaluations is presented by Ungar [2]; however, it does not offer guidance in terms of PBMs available to collect utility values for children. More recently, a set of good practice guidelines for measuring patient-reported outcomes in children and adolescents has been published by ISPOR, but again this does not specifically discuss existing PBMs [3]. Two recent systematic reviews have reviewed the use of PBMs for children in economic evaluations [4, 5]. We are not aware of any prior studies that have assessed the preferences of PBM responders (children and their proxies).

### Preference-based outcome measures (PBMs)

A PBM consists of a series of dimensions of health that are considered to be a determinant of a patient’s health-related quality of life (HRQOL). Patients rate these dimensions on a range of severity levels which are subsequently used to calculate a patient’s HRQOL in terms of their utility. Research has shown that dimensions of health that are considered important in adult populations are not the same dimensions that children consider important in terms of HRQOL. Children understand health in different ways than adults, with a greater interest in well-being and psychosocial health in contrast to adults that focus on the absence of a chronic illness/disability [2]. Therefore, the currently available adult PBMs are not directly applicable in child-based populations as they consist of dimensions that are not appropriate for children [6].

PBMs for children face several limitations in terms of design as well as practicality as they attempt to capture HRQOL information from a wide range of ages that are at various development stages. In terms of dimensions, it is difficult to have dimensions of HRQOL that are considered appropriate for infants, toddlers, and adolescents alike [6]. In addition, for the tool to be practical it must consider the linguistic and cognitive limitations of children at younger ages. This can be accomplished by incorporating visual images and presenting the information in a manner that children understand [2]. If the tool does not seek to accommodate younger patients but rather rely on proxy respondents, it should include observable events as it is difficult for proxy respondents to have a full understanding of non-observable dimensions.

### Obtaining responses to a PBM for children

PBMs are expected to be self-reported as they seek to quantify how health affects the patients’ HRQoL. However, the expectation of self-reporting poses difficulties in child-based populations due to cognitive and linguistic limitations, specifically for children under the age of 5 years. It should also be noted that while children aged 5–8 years may be able to self-report their health using appropriate measures, they tend to respond to measures with extreme reliance on “all or nothing” thinking which leads to responses on either extreme of the severity levels [2]. Previous literature on this topic suggests that patients of 0–5 years should have their HRQOL assessed by a proxy; patients 6–11 years should respond themselves, with questions read to them and the use of visual images if necessary; and 11–17-year-old children should be self-reporting [2].

Proxy respondents can include parents, guardians, teachers, and physicians. Studies that have examined the parent–child agreement when using parent proxies have found that correlations are highest in observable life dimensions (e.g., physical activities) and lowest in less observable domains (e.g., emotional functioning) [7].

When relying on proxy respondents there is a risk that parent–proxy respondents may over- or underestimate their child’s HRQOL; their response may be influenced by assumptions regarding their child’s potential treatment response, or their own health profiles and beliefs [8]. In an attempt to reduce this risk it has been recommended that a parent–child dyad be used, in which the parent and child respond to the questionnaire together with an interview guide who facilitates discussion in order to better represent the child’s response. When the parent–child dyad was used with the HUI2 and HUI3, it was determined that while there was no significant agreement between parent and child responses when obtained separately, there was moderate agreement between the child and the parent–child dyad responses on the HUI2 and HUI3 (0.55 and 0.74, respectively) [9].

The aim of this paper is to determine the experiences and opinions of child and proxy responders in the UK with respect to PBMs, to further inform the limited research on when and how child-appropriate PBMs should be applied in economic evaluation. We will synthesize the available literature on PBMs for child-based populations and summarize when it is appropriate for children and/or proxy respondents to be responding to the PBM. To achieve this we will:

1. Describe currently available PBMs for children in terms of their dimensions, severity levels, and valuation methods used.
2. Review current practice in the health economic literature to determine which tool is most frequently used and when patient and/or proxy respondents have completed the PBM in practice.
3. Present child and proxy preferences in responding to a PBM, obtained through a focus group attached to an ongoing clinical trial.
4. Outline and discuss key issues to consider when choosing a PBM for a child-based population, as well as when it is appropriate for children and/or proxy respondents to respond to the PBM.

## Methods

### Literature review

A literature review was conducted to determine current practice in terms of which tool is most frequently used for child-based economic evaluations and whether child or proxy responses are used. The search strategy used by Griebsch et al. was repeated in Medline and Embase (Table 1) [10] and papers were screened for inclusion by one author. The search term “utility” was also used to identify articles in the Paediatric Economic Database Evaluation [11]. The inclusion criteria were studies that collected utility values from child-based populations using direct or indirect methods. Studies that did not include primary collection of utility values, such as reviews, were excluded. The search was initially conducted in 2014 to inform the focus group discussions, and was updated in March 2016, including all papers published online by that date.

**Table 1:** Search strategy used in [10]

1. Infant, newborn/
2. Infant/
3. Child, preschool/
4. Child/
5. Adolescence/
6. 1 or 2 or 3 or 4 or 5
7. Exp quality-adjusted life-years/
8. (Cost utility or cost utility).mp (mp_ti, sh, ab, it, tn, ot, dm, mf, rw)
9. (Cost effectiveness of cost effectiveness).mp (mp_ti, sh, ab, it, tn, ot, dm, mf, rw)
10. 7 and 9
11. 8 or 10
12. 11 and 6

The currently available instruments were compared in terms of dimensions, severity levels, the elicitation and valuation methods used for the UK value set, the availability of country specific value sets, whether validation studies have been undertaken, and the range of possible utility values generated.

### Focus group

A series of five focus groups of proxy respondents and children were convened to determine patient preferences when responding to a PBM for child-based populations and which measure they preferred in terms of the EQ-5D-Y or CHU-9D. Focus groups are an ideal method when exploring subjects’ own meanings of health and illness-related concepts and for facilitating and observing co-construction of these meanings between participants. Children and parent–proxy respondents agreed to participate in a focus group on a variety of issues regarding the study design for an RCT on the early use of antibiotics for at-risk children with influenza in primary care (ARCHIE). For this study, “at-risk” children have been defined as children with underlying medical conditions or risk factors associated with an increased likelihood of developing influenza/ILI-related complications (e.g., asthma, congenital heart disease, or diabetes).

We conducted five focus groups in locations around the UK, the first of which was a pilot focus group to test and assess the interview guide and structure. All the focus groups were audio recorded (with written consent) and summarized in writing (not fully transcribed). Four of the focus groups, including the pilot, consisted of parents or carers of at-risk children, and one was a Medicines for Children Research Network (MCRN) panel of participants aged 11–20, mostly with long-term health conditions. All the focus groups were asked for feedback on the ARCHIE trial design, including the practical aspects of the trial participation process and the trial materials (e.g., study diaries and the PBM tool). During the focus groups, the participants were informed that the PBM selected would need to be completed in its entirety at days 1, 4, 7, 21, and 28 in a 28-day trial by either the child or a proxy respondent.

The focus groups were presented with the EQ-5D-Y and CHU-9D and were asked which tool they preferred and why, as well as who they felt should be responding to the PBM. The HUI2 was not presented to the focus group as prior discussion with the pilot focus group had found the HUI2 to be lengthy and overburdensome when collecting the instrument five times over a 4-week period.

## Results

### Literature review

Our literature review identified 941 articles, 109 of which met the inclusion criteria of obtaining utility values from child-based populations based on title and abstract. After full article review, we identified 45 studies that used a PBM for child-based populations. Many of the excluded studies included utility values cited in the literature that had been obtained from adult populations in other studies or used non-preference valuation techniques such as the VAS. Of the 45 included studies, 40 used a generic PBM tool, while five used only other direct elicitation methods (SG/TTO). In addition to cost–utility analyses, seven of the studies were methods papers, either mapping studies or measure development studies. A further 12 were valuation studies. A summary of the papers is included in the online appendix.

We identified five available PBMs that have been used for obtaining utility values for child-based populations: the Health Utility Index 2 (HUI2) and Health Utility Index 3 (HUI3), the Child Health Utility 9D (CHU-9D), the EuroQol youth version (EQ-5D-Y), and the Assessment of Quality of Life (AQOL-6D). While the HUI2 and CHU-9D have both been validated to be used for child-based populations in the UK, the EQ-5D-Y, HUI3, and AQOL-6D currently lack value sets. The EQ-5D-Y has been used with the UK adult valuation tariff as there is currently no EQ-5D-Y value set available. The HUI3 and AQOL-6D have no applicable value set in the UK and therefore cannot be used at the moment in the UK. While this study is from the UK perspective, tools without a UK value set have been included in the table below as there are value sets available for other countries (Table 2). The advantages and disadvantages of the different PBMs are presented below and summarized in Table 3.

**Table 2:** PBMs for child-based populations

(1) Author, year (2) Journal (3) Tool (4) Range of values	(1) Dimensions included (2) Severity levels	(1) Elicitation method (2) Valuation method	1) Validation (2) Countries with value sets
(1) Stevens (2012) (2) PharmacoEconomics (3) CHU-9D (4) 0.3369–1 (recalibrated for dead = 0 and perfect health = 1)	(1) Nine dimensions: worried, sad, pain, tired, annoyed, schoolwork, sleep, daily routine, and ability to join in activities (2) Five severity levels: no pain, little bit of pain, bit of pain, quite a lot of pain, a lot of pain	(1) Standard gamble (2) To establish the UK value set, 300 adults were interviewed in the UK using standard gamble methodology. Following the exclusion criteria, 282 respondents were included in the data analysis which equated to 2478 observations. The 300 respondents were interviewed face to face. The utility values were obtained from the UK general public. The public were not aware that they were valuing health states for children	(1) 6–17 years (2) UK; Australia
(1) McCabe [12] (2) Health economics (3) HUI 2 (4) − 0.064 to 1 (recalibrated for dead = 0 and perfect health = 1)	(1) Six dimensions: sensation, mobility, emotion, cognitive, self-care, and pain (2) 4–5 levels: Free of pain, occasional pain, frequent pain, frequent pain; frequent disruption of normal activities, severe pain	(1) Standard gamble (2) 20 face-to-face interviews were conducted in the UK among the UK general public. 198 interviews were conducted, with 176 interviews included in the data analysis after exclusion criteria had been applied. Those interviewed were told to imagine that they were a 10-year-old child and that they should expect to live the next 60 years	(1) Self-administered (12–18); interviewer-administered (8–18) (2) Canada, UK
(1) Wille [13] (2) Quality of life research (3) EQ-5D-Y (4) − 0.594 to 1 (recalibrated for dead = 0 and perfect health = 1)	(1) 5 dimensions: mobility, self-care, usual activities, pain/discomfort, anxiety/depression (2) Three severity levels: no pain, some pain, a lot of pain	No child-specific value set currently exists for the EQ-5D-Y. Therefore the responses are valued using the EQ-5D adult tariff. The following information is regarding the adult tariff (1) TTO (2) Face-to-face interviews were conducted in the UK among the UK general public. 3395 interviews were conducted, with 2997 interviews included after exclusion criteria. The valuation for the 13 states were elicited using a specially designed double-sided board. One side was relevant for states that were regarded as better than dead, and other side for states that were regarded as worse than being dead. Respondents were led by a process of “bracketing” to find their point of indifference between the two alternatives	(1) EQ-5D-Y has been used in children 7–18 years; however, it has not been validated (2) EQ-5D-3L adult value sets: Belgium, Denmark, Finland, France, Germany, Japan, New Zealand, Netherlands, Slovenia, Spain, UK, USA, Zimbabwe
(1) Moodie [14] (2) Health and quality of life outcomes (3) AQOL-6D Adolescent (4) Ranges vary by country: AQoL-6D Adolescent = (AQoL-6D adult)^1.19^, where 1.19 is the correction factor for Australia, 1.57—Fiji, 0.87—NZ & 1.87—Tonga	(1) Six dimensions: independent living (household tasks, mobility, walking, self-care), mental health (despair, worry, sadness, agitation), coping (energy, control, coping), relationships (friendships, family, community), pain (frequency of pain, degree of pain, effect on usual activities), senses (seeing, hearing, communication) (2) 4–5 Severity levels: pain interferes with usual activities never/rarely/sometimes/often/ always	(1) TTO (2) Surveys were administered in a classroom setting of students in Australia, New Zealand, Fiji, and Tonga. 279 secondary school students in total responded to the survey, with at least 60 students from each country. The 2790 health states valued were used to estimate the remaining health states using a multiplicative model	(1) 12–18 (2) Australia, New Zealand, Fiji, Tonga

**Table 3:** Advantages and disadvantages of PBMs for child-based populations

PBM	Advantages	Disadvantages
HUI2	UK value set available Large number of dimensions Self- or interviewer-administered Validated for children to self-report aged 9 and older	Difficult for some children to understand
HUI3	Large number of dimensions Self- or interviewer- administered Validated for children to self-report aged 9 and older	No UK value set available Difficult for some children to understand
EQ-5D-Y	Concise and simple to administer Well understood by researchers	No value set available Contains dimensions inappropriate for children
AQOL-6D	Adolescent-specific measure	No UK value set available
CHU-9D	UK value set available Validated for use in children aged 6 and older	Mental health components make it difficult for a proxy to complete UK valuation was from the perspective of adults

### PBMs for child-based populations

#### HUI

The HUI is the collective name for a pair of PBMs, the HUI2, and HUI3, which were originally developed for pediatric oncology patients but have been adapted as generic PBMs by excluding the fertility dimension. The HUI can either be self-administered (15Q) or interviewer-administered (40Q). If self-administered, it has been validated for self-assessment by children aged 12 and up, if interviewer-administered the HUI has been validated for self-assessment by children aged 9 and up [15]. The HUI2 consists of six dimensions and has four or five varying severity levels, depending on dimension, which were designed to measure levels of capacity based on abilities/disabilities. The HUI3 was developed to address the limitations of the HUI2, and consists of eight dimensions with five or six varying severity levels. The dimensions included were selected by a random sample of adults from the general population from a set of 15 domains that are considered key measures of health status [16]. The original value set was completed using a visual analogue scale in Canada; however, as part of the United Kingdom Pediatric Intensive Care Outcomes Study (PICOS), a UK valuation algorithm for the HUI2 was constructed using standard gamble methodology in adults imagining they were 10-year-old children, described in Table 2 [12].

In that study, 1370 utility values were obtained and were subsequently used to predict the remaining utility values using OLS regression. A total of 8000 health states were valued (with the exclusion of fertility) with a mean value ranging from − 0.064 to 0.79, with − 0.064 as the ‘PITS’ value [12].

Previous research has found that child respondents have reported problems with completing the HUI due to difficulty in understanding the questions, including the dimension and severity levels. Oluboyede et al. reported a higher amount of missing data for the HUI2 and HUI3 (ranging from 4 to 16% across all questions) than for the EQ-5D/EQ-5D-Y due to understanding difficulties with the HUI2 and HUI3 during a pilot study with adolescents aged 11–17 years, especially the questions related to ‘cognition’ in the HUI3 [17].

#### CHU-9D

The CHU-9D is a PBM that has been developed for children aged 7–11. It has also been validated for use in children aged 6–7 years and in adolescents aged 11–17 [18–20]. The CHU-9D is a generic measure and has nine dimensions with five levels of severity, with dimensions selected based on interviews with healthy school children aged 7–11 that elucidated what the subjects considered important aspects of health-related quality of life [6]. The UK valuation algorithm was constructed using standard gamble methodology in adults. As the valuation was intended to be representative of the general public, participants were not made aware that they were valuing health states for children (Table 2) [21].

Two thousand four hundred and seventy-eight utility values were obtained. The remaining health states were estimated using OLS parsimonious regression models. In order for all estimates to be statistically significant, some levels did have to be collapsed to have the same values. The survey results and the model results were combined to estimate a total of 1,953,125 possible health states which resulted in mean utility values ranging from 0.38757 to 0.931579, with a ‘PITS’ value (worst state as described by valuation questions) of 0.3368 and no states considered worse than death. The high ‘PITS’ value may be a result of adults taking an adults perspective on the more emotional elements and ultimately undervaluing the impact of mental health components on the HRQOL of children [21].

The CHU-9D was also valued by 590 adolescents in Australia using a discrete choice experiment (DCE) with best–worst health scaling among adolescents. Best–worst health scaling was considered more appropriate as it could be considered inappropriate to have children contemplate death, which would be required if using standard gamble or time trade off methodology. When comparing the results obtained from the UK general public value set and the Australian adolescent value set, it was found that the adult utility values are higher than those of adolescents, specifically in the mental health dimensions. Therefore it is possible that adolescents place more value on mental health dimensions than is suggested by the UK value set [22].

#### EQ-5D-Y

The EQ-5D-Y (Youth) is a modified version of the EQ-5D for children aged 7–18. The EQ-5D-Y has been tested for its feasibility, reliability, and validity; however, it is currently not recommended to be used by NICE as there is no value set specifically for children. Similar to the EQ-5D, the EQ-5D-Y has five dimensions and three severity levels. These dimensions were not altered as they were found to be applicable to children [13]. The five dimensions included in the EQ-5D-Y are mobility, looking after oneself, doing usual activities, having pain or discomfort, and feeling worried/sad/unhappy. These wording changes were implemented to enhance comprehensibility, with definitions changed for mobility (“walking about”) and looking after oneself (“washing/dressing oneself”), for example [13]. The severity levels were also altered to less extreme statements such as “a bit,” “some,” and “a lot.” During the development stage, children reported difficulties in understanding the self-care dimension of the EQ-5D-Y [13], and during reliability testing high ceiling effects became apparent in the mobility dimension with consistent responses of “no problems” [23].

While there is no UK value set specifically for the EQ-5D-Y, it has been used with the EQ-5D adult value set. To establish the UK EQ-5D adult value set, 3395 interviews were conducted in the UK. Following the exclusion criteria, 2997 interviews were included and valued using time trade off methodology (Table 2) [24].

In practice, proxy respondents have found the EQ-5D-Y dimensions inappropriate for children aged 4–7, specifically in terms of the mobility, self-care, and usual activities dimensions [25]. In addition, Canaway piloted the EQ-5D-Y and found that when using the adult EQ-5D-3L value set with the EQ-5D-Y, the adult value set resulted in states worse than death for children that were considered well enough to be in school at the time [18]. Additional work has been undertaken more recently exploring methods to be used to determine a valuation set for the EQ-5D-Y using adults in the USA [26].

#### AQOL-6D adolescent

The AQOL-6D adolescent is a PBM that has been recalibrated to be used with adolescents aged 12–18 as part of the Pacific Obesity Prevention in Communities (OPIC) project. While it was originally designed for use in obesity studies, it has since been used as a generic measure. It has six dimensions with five levels of severity [27]. There is no UK value set, but the existing Oceanian value set was valued in adolescents using time trade off methodology (Table 2) [14]. The 2790 health states valued were used to estimate the remaining health states using a multiplicative model. The mean utility scores obtained were 0.574 from students in Tonga and 0.799 from students in Australia [14].

### Current practice in economic evaluations for child-based populations

The most frequently used PBMs for child-based populations were the EQ-5D adult version and the HUI3. In total, 28.9% of studies collected a PBM that has been validated for use in child-based populations, with a majority of those using the HUI2. This translates to 21% of the total number of tools used, as 15 studies collected more than one PBM (Table 4). One study used the EQ-5D for children aged 4 and over, with PedsQL, a non-preference-based measure, mapped to the EQ-5D for children aged 2–4 years. 16 studies reported missing data (ranging from 0.7 to 49.3%).

**Table 4:** Current practice in economic evaluations for child-based populations (tools)

Tool	Number	% of studies using instrument (out of 45)	Age range	% of missing data reported (min–max across studies, where reported)
CHU-9D	4	8.9	6–17 years	0.5
EQ-5D	18	40	4–20 years	1–39.1
EQ-5D-Y	3	6.6	11–18 years	1.45–49.3
HUI2	9	20	0–18 years	3–27
HUI3	14	31.1	0–18 years	0.7–31
SF-12 (mapped to SF-6D)	1	2.2	9–16 years	16
SG	5	11.1	7–18 years	–
TTO	4	8.9	4–18 years	27
Total	58			

**Fig. 1:**
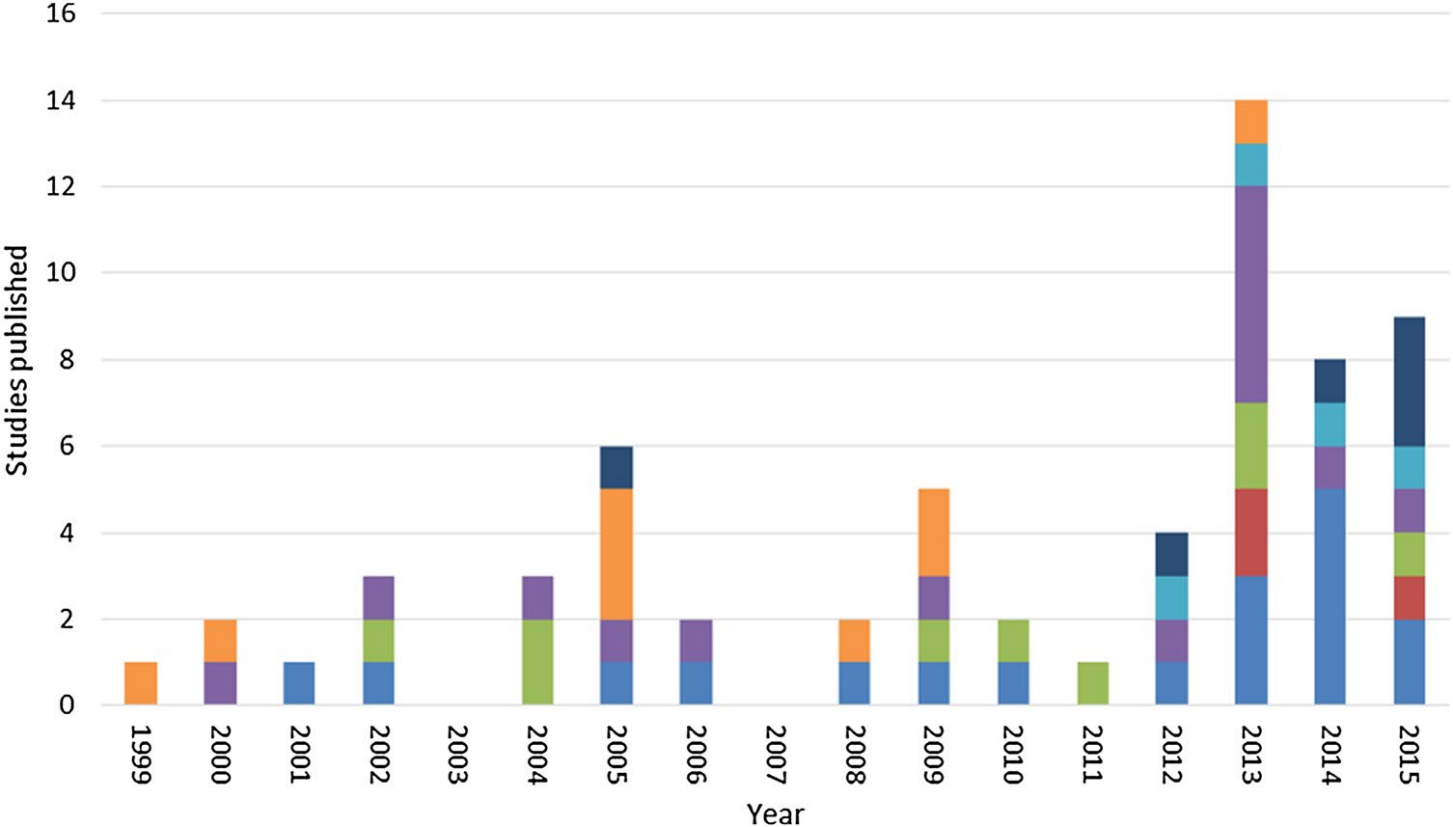
Trends in the use of PBMs over time. Dark blue bar—EQ-5D, red bar—EQ-5D-Y, light green bar—HUI2, violet bar—HUI3, light blue bar—CHU-9D, orange bar—Direct, navy blue bar—Other. (Color figure online)

**Fig. 2:**
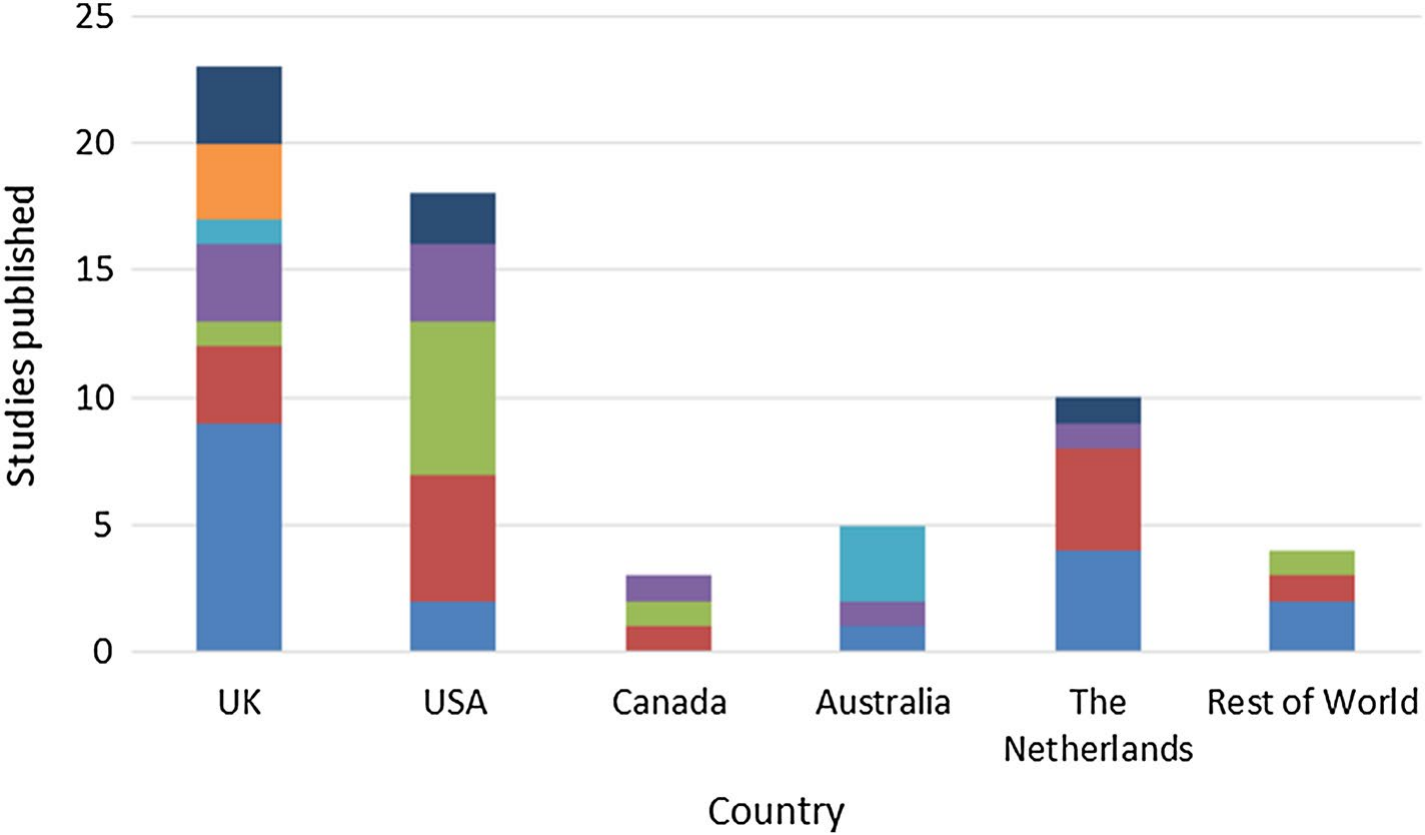
Choice of PBM by country. Dark blue bar—EQ-5D, red bar—HUI3, light green bar—Direct, violet bar—HUI2, light blue bar—CHU-9D, orange bar—EQ-5D-Y, navy blue bar—Other. (Color figure online)

There has a been a significant increase in the number of studies collecting utility values from child populations in recent years, but there has been very little corresponding increase in the use of child-specific PBMs. Most studies published since 2011, the first year the EQ-5D-Y was used in our review set, have used either the EQ-5D adult version or HUI3 (Fig. 1). Choice of PBM is consistent across countries, with the EQ-5D, HUI3, and direct methods all commonly used, although child-specific PBMs are most frequently used in the UK, and in Australia the CHU-9D is used in half of all studies (Fig. 2).

**Fig. 3:**
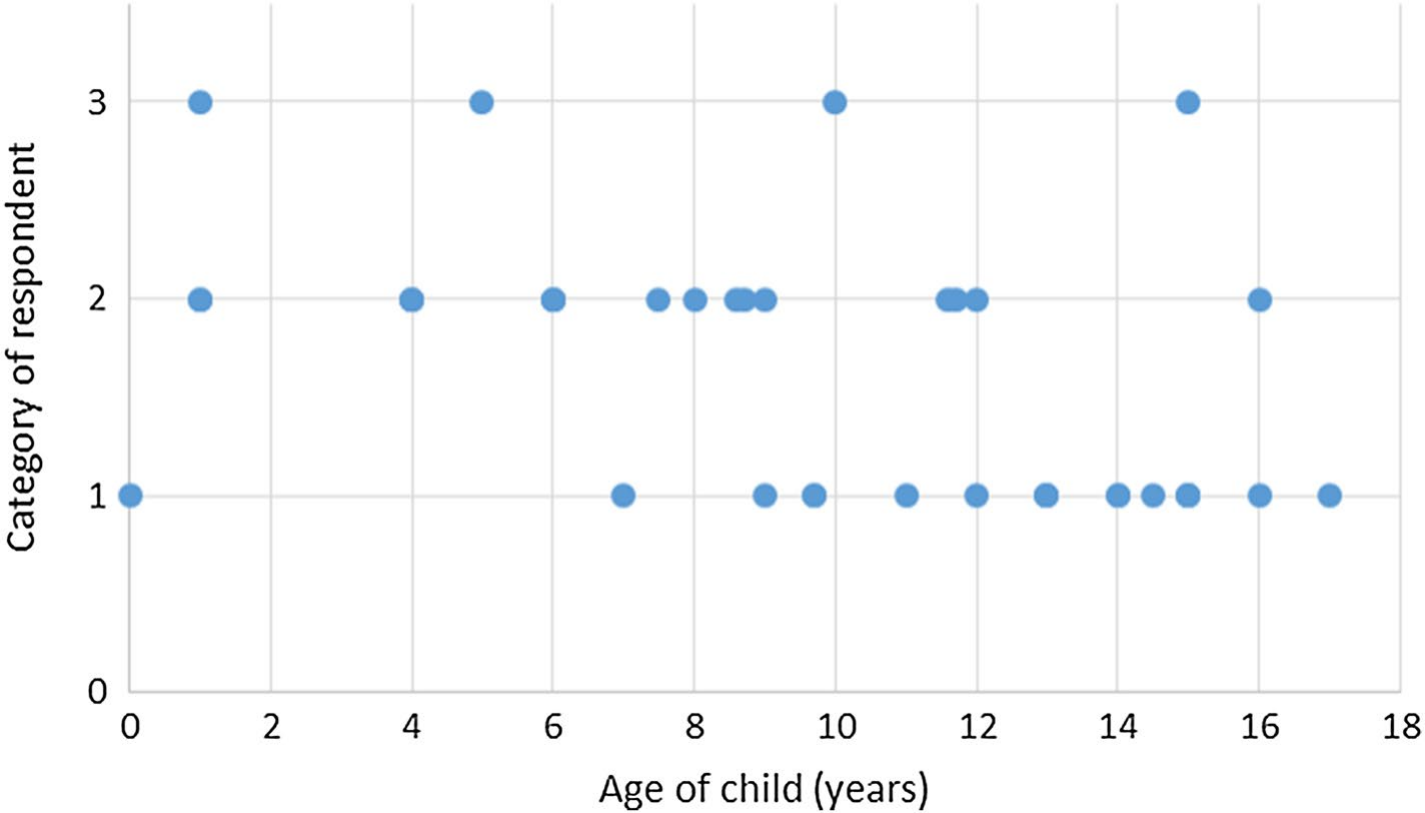
Respondent by average child age. *1* Child, *2* Parent–proxy, *3* Unclear. Ages are means or medians, unless only a range is reported, in which case the middle of the range was used

In terms of who is responding to the PBMs, current practice suggests that parent–proxy respondents are the most common respondents. This is to be expected of studies completed with infants and young children; however, parents were also proxy respondents for children up to 18 years of age (Fig. 3). The youngest children self-reporting were 6 years of age. Four studies did not clearly report who the respondent was, although this could usually be inferred from the age of participants, as these studies often used either very young participants or adolescents. One study implied that a child–parent dyad was used, but this was not clearly reported [28]. Physician responses were used only as expert opinion for general valuations for health states, rather than as proxies for a specific individual children.

These findings are similar to those found by Kromm et al. which identified cost–utility analyses in the Pediatric Economic Database Evaluation (PEDE) and included studies with utility/disutility values [29]. They also agree with more recent reviews, which found that proxies are the most frequent respondents, and that the EQ-5D, despite not being recommended for use in child populations, is one of the most commonly used PBMs [4, 5].

### Focus group

As part of this study, we sought to determine patient preferences in responding to a PBM as part of a clinical trial and economic evaluation. The focus groups were convened consisting of patients and parents who fulfilled the inclusion criteria for the ongoing ARCHIE study which explores the use of Antibiotics in ‘at-risk’ children with influenza-like illness [30].

Five focus groups were convened at sites around the UK, four (one of which was a pilot) consisting of parents in Oxford, London, and Liverpool (21 participants in total), and one of young people aged 11–20 in Birmingham (15 participants).

The parent–proxy respondents found the EQ-5D-Y more applicable across different ages and underlying conditions, and noted the benefits of fewer questions. In contrast, they felt the CHU-9D captured more dimensions of health, and may therefore capture overall HRQoL more fully. One concern regarding the CHU-9D was that it would be difficult or even irrelevant to answer as proxy respondents for younger children (e.g., babies/toddlers) as it contained questions of the child’s emotional state, for example, sadness, worry, and annoyance.

Parents expressed a concern over the lack of sensitivity of both instruments. In particular, they were concerned that the use of the tools in an at-risk population may fail to differentiate between the symptoms of an acute illness and those of the child’s underlying condition. For example, in a disabled child unable to speak or walk, the questions would in fact pick up the disability rather than any deterioration in their health due to influenza-like illness. Parent–proxy respondents felt in general that they would be more appropriate respondents as children may have difficulty completing the questionnaires especially when ill.

Parents additionally reported confusion at whether their responses should reflect the overall quality of life of their child, including any pre-existing conditions, or should just relate to changes compared to usual that the participants felt were related to the health condition in question.

The MCRN young people’s focus group found that the EQ-5D-Y had a greater focus on tangible experiences and allowed straightforward responses, whereas the CHU-9D included more representative severity levels as there was a wider array of options. There was a diversity of opinion in the group by age, with older respondents preferring the CHU-9D for the additional detail it provided. The younger respondents in the focus group preferred the EQ-5D-Y, finding the CHU-9D difficult to understand; they felt they may need assistance in responding. In terms of responding for themselves or via a proxy, they expressed a concern with parents not being able to know exactly how they feel. Overall, the young people’s group expressed that they would appreciate the option to respond for themselves, although they were overall neutral to responding on their own behalf or by proxy.

## Discussion

Our review found that a child-based PBM must include dimensions that children consider an aspect of HRQOL and must also be age-appropriate for the respondent’s developmental stage. If a PBM includes dimensions that are considered irrelevant to children this could result in a high ceiling effect with patients reporting “no problems” as they do not consider it an element of HRQOL such as the ceiling effects related to the mobility dimension in the EQ-5D-Y [23]. If it includes dimensions that are not age-appropriate alternatively there could be a floor effect as the PBM is picking up dimensions that reflect a child’s development stage rather than HRQOL such as “unable to control arms or legs.” A recent comparison of the EQ-5D-Y and the CHU-9D found substantial differences in responses between the two instruments in poorer health states: among respondents with EQ-5D-Y scores ≤ 0.2 the mean CHU-9D score was 0.58 compared with a mean of 0.05 for the EQ-5D-Y [31]. Either the CHU-9D is not fully capturing worse health states, or the EQ-5D-Y is undervaluing some children’s health states, perhaps reflecting the difficulty of accurately capturing children’s well-being in an instrument that has been adapted from adults. For example, by placing more weight on dimensions like self-care, which may not substantially affect children’s true utility. Additionally, this paradigm raises the concern of the practicality of having one PBM that is applicable to all children from infancy through adolescence. Due to rapid changes in cognitive and physical abilities over this time, several instruments may be required to adequately capture HRQOL for this population rather than reliance on one generic PBM.

There are currently two PBMs designed for child-based populations with UK value sets: the HUI2 and the CHU-9D. The HUI2 consists of adult dimensions with a value set obtained from adults with children in mind, while the CHU-9D consists of child-specific dimensions with a value set obtained from adults without children in mind. The EQ-5D-Y is currently not recommended to obtain utility values for child-based populations as it contains a descriptive system developed so that it can be understood by children and adolescents, currently with no corresponding value set. This however is in development, with value sets obtained from adults with children in mind. It remains unclear how the instruments to collect utility values for child-based populations should be designed.

There is no consensus on how the PBMs should be valued. If the PBMs for child-based populations are to be valued with children in mind as evidenced by the findings of Kind and colleagues [32], consideration may need to be taken regarding the introduction of age bias into the valuation process. If valuation should be completed by children, the option for valuation by discrete choice experiment among adolescents is also a possibility, or with best–worst scaling, which is cognitively easier and eliminates the need for children to contemplate death.

While the HUI2 and the CHU-9D are available and fit for purpose, many health economic evaluations with child-based populations have instead obtained utility values using the EQ-5D adult version or the HUI3. This may be reflective of practicality as the HUI2 may be considered difficult to understand, which could result in a high amount of missing data. This is supported by the results from the focus groups, which rejected the HUI2 as being overly long. An alternative explanation, given the widespread use of the HUI3, is that researchers are more familiar with the EQ-5D and HUI3 from evaluations in adult populations, and therefore choose to use the more familiar instrument, given the lack of value sets and guidance surrounding the use of these more specialized PBMs. The greater use of the EQ-5D could also reflect patient preferences towards the EQ-5D/EQ-5D-Y. This is supported by our focus group, who found the EQ-5D-Y easier to complete and more straightforward than the CHU-9D. It could also reflect necessity, as both the HUI2 and CHU-9D only have two available value sets, while the EQ-5D-3L adult version has 13 value sets. This may explain the widespread use of the CHU-9D in Australia, where a value set using adolescent valuations is available.

In terms of responding to a PBM, little evidence exists on this matter. Current practice suggests that proxy respondents are the most frequent respondents. While proxy respondents are often required to respond on behalf of children simply due to cognitive limitations, proxy respondents are also being used for children aged 18 in current practice. Not including the child’s own responses raises the possibility of misrepresentation due to the presence of unobservable dimensions.

This review adds to the existing literature by building on previous reviews in this area, and by providing qualitative evidence on the views of children and their proxies with respect to the choice of PBM used. However, there are a number of limitations to the validity of the results. One is that the focus groups were convened primarily for the purpose of informing the design of the ARCHIE trial. As a result, the participants were patients with conditions that increased their risk of developing complications associated with influenza. Although this covers a broad range of patients, it may limit the generalizability of the results. Additionally there was no formal qualitative analysis of the focus groups, all were audio recorded (with written consent) and summarized in writing but not fully transcribed, Finally, only a single reviewer conducted the literature searches, so some studies may have been missed.

### Guidance for using PBMs in children

Our focus group results raise important considerations for researchers who use PBMs in child populations. Researchers should give significant consideration to who responds to the PBM. Most previous studies have used parent–proxy respondents, but while parents in our focus groups preferred to be involved in responding, they also recognized that unobservable characteristics in PBMs limited the value of their proxy responses. All PBMs we have reported on include unobservable events. Therefore, attempts should be made to allow children as young as the age of 5 to respond, with either assistance from the proxy or through a parent–child dyad approach.

The children in our focus groups expressed a preference for responding on their own behalf, although parental input was welcome for younger children. Older children and adolescents had a clear preference in our focus group for responding on their own behalf.

The age of the children in the study will also affect the choice of PBM, with younger children preferring the simpler, shorter EQ-5D-Y over CHU-9D. Similarly, the HUI2 was eliminated from our focus groups at the pilot stage, due to a clear preference against such an extensive PBM, particularly where it will be completed multiple times.

Finally, the PBM value sets are drawn from a variety of different perspectives. The EQ-5D-Y does not currently have a corresponding preference weight-based value set. The CHU-9D used a UK adult general population for its UK value set and an Australian adolescent population for its Australian value set. The HUI2 used adult members of the UK population imagining they were 10-year-old children, while the AQOL-6D used healthy adolescents. Given the obvious weaknesses associated with assigning adult preferences to children, careful consideration should be given to the most appropriate method for valuing children’s health states.

## Conclusion

NICE currently does not specify which PBM should be used to obtain utility values for child-based populations nor does it clarify when it is appropriate for patients or proxy respondents to respond to the PBM. This study aimed to synthesize the available literature on obtaining utility values for child-based populations in terms of PBMs that are available to use and who should be responding to those PBMs. We identified 2 PBMs that are appropriate to be used in child-based populations with a UK value set: the HUI2 and the CHU-9D. In addition, the literature suggests that patients aged 5–18 can participate in responding to a PBM with assistance from a proxy respondent if necessary. However, in current practice the EQ-5D is the most frequently used PBM in child-based populations, and parent–proxy respondents are the most frequent respondents.

Our study found that there are currently no age-appropriate PBMs for children under the age of 5 nor PBMs specifically designed for proxy respondents consisting of only observable events.

Comparing the focus group findings with the results of the review demonstrates that there is a disconnect between the preferences of participants and the choices of PBMs and respondents made by researchers in real-world studies, in particular, the reliance on parent–proxy respondents over the input of children/adolescents themselves, despite all PBMs containing unobservable dimensions.

## Electronic supplementary material

Below is the link to the electronic supplementary material.

Supplementary material 1 (DOCX 60 KB)

## Abbreviations

PBM: Preference-based measure
EQ-5D-Y: EuroQol 5-dimension youth
SF-36: Short-form 36
CHU-9D: Child heath utility 9 dimension
HUI Health: Utilities index
AQOL-6D: Assessment of quality of life 6 dimension
