# Supplementary material for: Preference-based measures to obtain health state utility values for use in economic evaluations with child-based populations: a review and UK-based focus group assessment of patient and parent choices
Source: Qual Life Res. 2018 Mar 21;27(7):1769–80. doi: 10.1007/s11136-018-1831-6 (PMC5997719; doi:10.1007/s11136-018-1831-6)
Supplement: Supplementary file 1 — Supplementary material 1 (DOCX 60 KB) [file 11136_2018_1831_MOESM1_ESM.docx]

Summary of papers collecting health state utility values in economic evaluations

|  | 1) First Author  2) Journal  3) Date  4) Country | 1) Study Aim | 1) age of patients  2) respondent (Child, Parent, Physician)  3) tool/method used  4) Missing data (if any missing data was reported) |
| --- | --- | --- | --- |
|  | 1. Anderson 2. Journal of Child Psychology and Psychiatry 3. 2014 4. UK | To estimate the cost-effectiveness of classroom-based cognitive behavioural therapy using trial-based cost-utility analysis. | 1. 12 – 16 years 2. Child 3. EQ-5D 4. 38% |
|  | 1. Arnold 2. Biology of Blood and Bone Marrow Transplantation 3. 2015 4. USA | To estimate the cost-effectiveness of allogeneic haematopoietic cell transplantation in children with sickle cell disease using a cost-utility analysis | 1. <21 years 2. Child 3. EQ-5D |
|  | 1) Barton  2) Ear & Hearing  3) 2006  4) UK | To estimate the cost-effectiveness of paediatric cochlear implantation by conducting a cost-utility analysis. | 1) Not clearly stated  2) parent-proxy respondent  3) HUI3  4) 31% |
|  | 1) Brisson  2) The Paediatric Infectious Disease Journal  3) 2010  4) Canada | To describe the impact of rotavirus associated gastroenteritis on HR-QOL, more specifically on estimating the QALY-lost to RGE in children and their parents for use in the cost-effectiveness of rotavirus vaccines. | 1) < 36 months  2) parents-proxy respondent  3) HUI2 |
|  | 1) Canaway  2) Quality of Life Research  3) 2013  4) UK | To examine the performance of CHU-9D and EQ-5D-Y in children aged 6-7 years. | 1) 6-7 years  2) Child  3) CHU-9D, EQ-5D-Y |
|  | 1) Carroll  2) The journal of paediatrics  3) 2009  4) USA | To gather and calculate utilities for a wide range of health states in the paediatric population. | 1) <18 years  2) parent-proxy respondent  3) TTO, SG |
|  | 1) Chen  2) Jama Dermatology  3) 2008  4)USA | To measure acne-related health state utilities among adolescents by assessing health outcome preferences. | 1) 14-18 years  2) child  3) TTO |
|  | 1. Chen 2. Health and Quality of Life Outcomes 3. 2014 4. Australia | To develop an algorithm for mapping KIDSCREEN-10 to CHU-9D in an adolescent population. | 1. 11 – 17 years 2. Child 3. CHU-9D |
|  | 1) Cheng  2) JAMA  3) 2000  4) USA | To determine the quality of life and cost consequences for deaf children who receive a cochlear implant. | 1) 10 years  2) parent-proxy respondent  3) TTO, HUI3  4) 27% |
|  | 1) Chiou  2) International Journal for Quality in Health Care  3) 2005  4) USA | To develop a multi-attribute outcome measure for children with asthma that allows for the calculation of quality adjusted life years in cost-effectiveness studies and also to assign preference weights asthma-symptom free days. | 1) 4-12 years  2) Child  3) PAHOM, SG |
|  | 1. Clement 2. The Bone & Joint Journal 3. 2015 4. UK | The estimate the clinical and cost-effectiveness of prophylactic fixation of the contralateral hip in patients with unilateral slipped capital femoral epiphysis. | 1. 9 – 16 years 2. Child 3. SF-12 converted to SF-6D 4. 16% |
|  | 1) Connock  2) HTA  3) 2006  4) UK | To examine the clinical effectiveness and cost-effectiveness of newer antiepileptic drugs (AEDs) for epilepsy in children. | 1) 7-12 years  2) physician- proxy respondent  3) EQ-5D |
|  | 1. Creswell 2. HTA 3. 2015 4. UK | To determine the clinical and cost-effectiveness of child CBT alongside maternal CBT for maternal anxiety disorder, using a trial based evaluation. | 1. 6 – 13 years 2. Not clearly stated 3. EQ-5D-Y 4. 1.45 – 49.3% |
|  | 1. De Sonneville-Koedoot 2. Journal of Communication Disorders 3. 2015 4. The Netherlands | To estimate the cost-effectiveness of two interventions for stuttering in pre-school children using a trial-based evaluation. | 1. 3 – 6.3 years 2. Parent-proxy respondent 3. EQ-VAS/HUI3 |
|  | 1) Ekert  2) Haemophilia  3) 2001  4) Australia | To determine the clinical effectiveness of providing on-demand FVIIa therapy vs. their previous usual care; to determine QoL values with validated instruments; to determine cost-effectiveness by estimated cost-per-QALY. | 1) 11-16 years  2) Child  3) EQ-5D |
|  | 1. Furber 2. Health and Quality of Life Outcomes 3. 2015 4. Australia | To assess the suitability of CHU-9D as a routine outcome measure in CAMHS clinical practice for children and adolescents. | 1. 5 – 17 years 2. Parent/Adult relative proxy respondent 3. CHU-9D 4. 0.5% |
|  | 1) Greenough  2) Arch Dis Child  3) 2004  4) USA | To determine whether RSV hospitalisation in the first two years was associated with chronic respiratory morbidity during the preschool years in prematurely born children who had chronic lung disease. | 1) 5 years  2) parent- proxy respondent  3) HUI2, HUI3 |
|  | 1) Hollmann  2) PLOS One  3) 2013  4) Spain | To assess the changes in health-related quality of life in patients with confirmed diagnosis of influenza (H1N1) 2009, and to estimate the individual and societal loss of quality-adjusted life years caused by the pandemic. | 1) 8-17 years  2) parent- proxy respondent  3) EQ-5D  4) 19% |
|  | 1. Ising 2. Psychological Medicine 3. 2015 4. The Netherlands | To assess the cost-effectiveness of CBT added to standard care in preventing first-episode psychosis, using an RCT-based evaluation. | 1. 14+ years 2. Child 3. EQ-5D-3L |
|  | 1. Kanters 2. Orphanet Journal of Rare Diseases 3. 2014 4. The Netherlands | To assess the cost-effectiveness of enzyme-replacement therapy for infantile Pompe disease using a societal-perspective cost-utility analysis alongside an observational study. | 1. 2+ years 2. Parent-proxy respondent 3. EQ-5D |
|  | 1) Lee  2) health and Quality of Life outcomes  3) 2005  4) USA | To describe patient valuations of pertussis disease and vaccination and to compare valuations for short-term and long-term health states associated with pertussis. | 1) 11-17 years  2) parent-proxy respondent  3) TTO |
|  | 1. Little 2. HTA 3. 2014 4. UK | To assess the cost-effectiveness of strategies for improving appropriate use of antibiotics for sore throat, using a trial-based evaluation. | 1. >5 years 2. Not clearly stated 3. EQ-5D 4. 48% |
|  | 1. Marlow 2. Vaccine 3. 2015 4. UK | To assess the quality of life impact of rotavirus gastroenteritis on children and their families in the UK. | 1. <6 years 2. Not clearly stated 3. EQ-VAS/HUI2 |
|  | 1) Martin  2) Journal of Medical Economics  3) 2008  4) UK | To estimate utility scores for different severities of acute rotavirus gastroenteritis in children <5 years in the UK. | 1) <5 years  2) physician- proxy respondent  3) EQ-5D |
|  | 1. Payakachet 2. Autism Research 3. 2014 4. USA | To Develop mapping algorithms to enable utility scores to be derived from clinical and behavioural outcome measures in children and adolescents with autism. | 1. 4 – 17 years 2. Primary caregiver-proxy respondent 3. HUI3 4. 2.7% |
|  | 1) Oluboyede  2) European Journal of Health Economics  3) 2013  4) UK | To investigate problems with language and comprehension associated with the completion of the aforementioned HRQOL questionnaires. | 1) 11-17 years  2) child  3) EQ-5D, HUI2, HUI3, EQ-5D-Y  4) 10% for EQ-5D-Y; 27% for HUI2/HUI3 |
|  | 1) Oostenbrink  2) journal of clinical epidemiology  3) 2002  4) The Netherlands | To evaluate the quality weights for permanent sequelae after childhood bacterial meningitis obtained with two different classification instruments-the EQ-5D and HUI 3. | 1) Not clearly stated  2) physician-proxy respondent  3) EQ-5D, HUI2 , HUI3  4) 1% EQ-5D, 3% HUI2/HUI3 |
|  | 1. Petrou 2. International Journal of Technology Assessment in Health Care 3. 2014 4. UK | To estimate the cost-effectiveness of nebulized magnesium sulphate in acute asthma in children in the NHS. | 1. 5 – 16 years and 2 – 4 years 2. Parent-proxy respondents 3. EQ-5D and PedsQL mapped to EQ-5D for 2 – 4 year olds |
|  | 1. Philipsson 2. Cost Effectiveness and Resource Allocation 3. 2013 4. Sweden | To estimate the cost-effectiveness of a dance intervention for adolescent girls with internalizing problems, using a trial-based evaluation. | 1. 13 – 18 years 2. Not clearly stated (implied child) 3. HUI3 4. 17% |
|  | 1) Polsky  2) Addiction  3) 2010  4) USA | To estimate cost, net social costs and cost-effectiveness in a clinical trial of extended buprenoprhone-naloxone treatment versus brief detoxification treatment in opiod dependent youth. | 1) 15-21 years  2)child  3) EQ-5D  4) 39.1% |
|  | 1) Ratcliffe  2) Pharmacoeconomics  3) 2012  4) Australia | To apply profile case best-worst scaling (BWS) discrete-choice experiment methods to obtain adolescent-specific values for the CHU-9D. Also to assess the feasibility of a web-based method of data collection for the valuation of health states defined by the CHU-9D. | 1) 11-17 years  2) child  3) CHU-9D |
|  | 1) Rodriguez- Martinez  2) Journal of Asthma  3) 2013  4) Colombia | To estimate the incremental cost-utility ratio of three inhaled corticosteroids currently licensed in Colombia for use in children with persistent asthma, fluticasone propionate, and ciclesonide compared to beclomethasone dipropinoate. | 1) < 18 years  2) parent- proxy respondent  3) SG |
|  | 1) Saigal  2) Jama  3) 1999  4) Canada | To measure and compare preferences for selected health states from the perspective of health care professionals, parents of extremely low birth with or normal birth weight infants, and adolescents who were either extremely low birth weight or normal birth weight infants. | 1) 8-16 years  2) physician-proxy respondent  3) SG |
|  | 1) Secnik  2) Medical Decision Making  3) 2005  4) UK | To use standard gamble (SG) utility interviews to assess parent preferences for health states of childhood attention-deficit/hyperactivity disorder (ADHD). | 1) 7-18 years  2) parent-proxy respondent  3) SG, EQ-5D |
|  | 1. Semenov 2. Ear and Hearing 3. 2013 4. USA | To assess the consequences for cost-utility of the timing of cochlear implantation, using a prospective study-based evaluation taking a societal perspective. | 1. Not clearly stated (0 – 47 months, approx.) 2. Parent-proxy respondent 3. HUI2 and HUI3 |
|  | 1) Sruamsiri  2) BMC Health Services research  3) 2013  4) Thailand | To examine the potential cost-utility of RI-HSCT (reduced intensity hematopoietic stem cell transplantation compared to BT-ICT (iron chelating therapy) in treatment of patients with severe adolescent and young adult thalassemia. | 1) 9-18 years  2) Child  3) EQ-5D |
|  | 1) Tilford  2) Quality of Life Research  3) 2004  4) USA | To provide information on the preference scores of children with spina bifida aperta and to measure the impact of caring for a child with spina bifida. | 1) <18 years  2) parent-proxy respondent  3) HUI2 |
|  | 1) Tilford  2) Pharmacoeconomics  3) 2012  4) USA | To describe the HRQOL outcomes in children with autism spectrum disorders. | 1) 4-17 years  2) parent-proxy respondent  3) HUI3, QWB  4) 0.7%-2.7% |
|  | 1) Van den Akker-Van Marle  2) BJOG  3) 2005  4) The Netherlands | To compare the costs and effects of different alternative treatment strategies utilising intrapartum antibiotic prophylaxis to prevent early-only group B haemolytic streptococcal disease. | 1) 2-8 years  2) parent –proxy respondent  3) HUI3 |
|  | 1. Van Litsenburg 2. Quality of Life Research 3. 2013 4. The Netherlands | To assess health-related quality of life in survivors of acute lymphoblastic leukaemia in the short term (6 months to 4 years). | 1. ≥ 5 years 2. Parent-proxy respondent 3. HUI3 |
|  | 1. Van Steensel 2. Research in Autism Spectrum Disorders 3. 2014 4. The Netherlands | To investigate the cost-effectiveness of cognitive behavioural therapy as compared to usual care for anxiety in children with autism-spectrum disorders, using a trial based evaluation. | 1. Not clearly stated (mean 11) 2. Child/Parent-proxy respondent 3. EQ-5D 4. 6% |
|  | 1) Wen  2) Contemporary Clinical Trials  3) 2011  4) Australia | To determine if an early childhood obesity intervention in children aged up to 2 years will lead to lower mean BMI, lower screen time, improved dietary behaviours and demonstrated cost-effectiveness of the intervention in children aged 3 ½ to 5. | 1) 5 years  2) Not clearly stated  3) HUI2 |
|  | 1) Williamson  2) HTA  3) 2009  4) UK | To determine the clinical effectiveness of topical momestasone in children with otitis media with effusion in both ears. | 1) 4-11 years  2) parent-proxy respondent  3) The HUI2, HUI3, ED-5D-5L  4) 45% |
|  | 1) Wyatt  2) Health Technology Assessment  3) 2012  4) UK | To determine natural history and estimate effectiveness and cost of enzyme replacement therapy (ERT) and substrate replacement therapy (SRT) for patients for Gaucher disease, Fabry disease, mucopolyscaccharidosis type I and type II, Pompe disease and Nieumann-Pick type C disease. | 1) >13 years  2) child  3) EQ-5D |
|  | 1) Young  2) Journal of Paediatrics  3) 2013  4) Canada | To describe the health and health related quality of life outcomes of youths and young adults with spina bifida. | 1) 13-18 years  2) child  3) HUI3 |

Valuation based studies. Methodology based studies. Economic evaluation.
